# Enabling 2D Electron Gas with High Room‐Temperature Electron Mobility Exceeding 100 cm^2^ Vs^−1^ at a Perovskite Oxide Interface

**DOI:** 10.1002/adma.202409076

**Published:** 2024-10-22

**Authors:** Georg Hoffmann, Martina Zupancic, Aysha A. Riaz, Curran Kalha, Christoph Schlueter, Andrei Gloskovskii, Anna Regoutz, Martin Albrecht, Johanna Nordlander, Oliver Bierwagen

**Affiliations:** ^1^ Paul‐Drude‐Institut für Festkörperelektronik Leibniz‐Institut im Forschungsverbund Berlin e. V. Hausvogteiplatz 5–7 10117 Berlin Germany; ^2^ Leibniz‐Institut für Kristallzüchtung Max‐Born‐Straße 2 12489 Berlin Germany; ^3^ Department of Chemistry University College London 20 Gordon Street London WC1H 0AJ UK; ^4^ Deutsches Elektronen‐Synchrotron DESY 22607 Hamburg Germany; ^5^ Inorganic Chemistry Laboratory Department of Chemistry University of Oxford South Parks Road Oxford OX1 3QR UK

**Keywords:** BaSnO_3_, high mobility, LaInO_3_, molecular beam epitaxy, perovskite oxides, 2D electron gas

## Abstract

In perovskite oxide heterostructures, bulk functional properties coexist with emergent physical phenomena at epitaxial interfaces. Notably, charge transfer at the interface between two insulating oxide layers can lead to the formation of a 2D electron gas (2DEG) with possible applications in, e.g., high‐electron‐mobility transistors and ferroelectric field‐effect transistors. So far, the realization of oxide 2DEGs is, however, largely limited to the interface between the single‐crystal substrate and epitaxial film, preventing their deliberate placement inside a larger device architecture. Additionally, the substrate‐limited quality of perovskite oxide interfaces hampers room‐temperature (RT) 2DEG performance due to notoriously low electron mobility. In this work, the controlled creation of an interfacial 2DEG at the epitaxial interface between perovskite oxides BaSnO_3_ and LaInO_3_ is demonstrated with enhanced RT electron mobility values up to 119 cm^2^ Vs^−1^—the highest RT value reported so far for a perovskite oxide 2DEG. Using a combination of state‐of‐the‐art deposition modes during oxide molecular beam epitaxy, this approach opens up another degree of freedom in optimization and in situ control of the interface between two epitaxial oxide layers away from the substrate interface. Thus this approach is expected to apply to the general class of perovskite oxide 2DEG systems and to enable their improved compatibility with novel device concepts and integration across materials platforms.

## Graphical abstract

In this work, using a combination of state‐of‐the‐art modes in molecular beam epitaxy, the controlled creation of a 2D electron gas at the epitaxial interface between perovskite oxides BaSnO_3_ and LaInO_3_ is demonstrated. The enhanced room‐temperature electron mobility values are up to 119 cm^2^ Vs^−1^—the highest room‐temperature value reported so far for a perovskite oxide 2D electron gas.

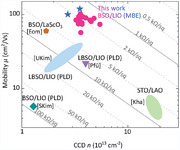

## 1 Introduction

The family of complex oxides comprises dielectric, semiconducting, superconducting, ferromagnetic, and ferroelectric materials. The wide range of functionalities hosted by especially the perovskite *AB*O_3_ oxides, in combination with the possibility of integration on silicon,^[^ ^1^ ^]^ motivates the realization of oxide electronic heterostructures with precision on par with established semiconductor thin‐film synthesis. By interfacing such materials epitaxially, new (multi)functional heterostructures can be constructed exhibiting physical phenomena beyond bulk properties.^[^ ^2^, ^3^ ^]^ Notably, oxide interfaces, representing 2D objects within a multilayer structure, can give rise to 2D electron gases (2DEGs),^[^ ^4^ ^]^ superconductivity,^[^ ^5^ ^]^ interface magnetism,^[^ ^6^ ^]^ and Rashba spin‐orbit coupling.^[^ ^7^ ^]^ In the case of 2DEGs, non‐polar SrTiO_3_ (STO) interfaced with other formal‐polar perovskites (exhibiting charged lattice planes), such as LaAlO_3_,^[^ ^4^, ^8^, ^9^ ^]^ LaTiO_3_,^[^ ^10^ ^]^ or GdTiO_3_,^[^ ^11^, ^12^ ^]^ served as a work‐horse for polarization‐discontinuity‐doped 2DEGs that pushed the evolution of exotic physical phenomena such as spin‐charge conversion,^[^ ^13^ ^]^ and 2D superconductivity.^[^ ^5^ ^]^ These systems suffer, however, from a low STO‐related room‐temperature (RT) electron mobility (μ_RT_ < 10 cm^2^Vs^−1^)^[^ ^9^, ^14^ ^]^ limiting their applicability for most device types.

Other oxide systems have been proposed to realize higher RT 2DEG mobility. Notably, Sn‐based systems show particular promise, as their conducting channel is formed by highly delocalized Sn s states leading to a lower effective electron mass compared to the prototypical STO‐based heterostructures relying on more localized Ti 3d orbitals.^[^ ^15^, ^16^, ^17^ ^]^ One such system is based on BaSnO_3_ (BSO), a wide‐bandgap semiconducting oxide with the highest reported bulk (3D) RT electron mobility of up to 320 cm^2^ Vs^−1^ in single crystals,^[^ ^18^ ^]^ and 120 ‐ 180 cm^2^ Vs^−1^ in La‐doped BSO (LBSO) thin films grown by molecular beam epitaxy (MBE).^[^ ^19^, ^20^, ^21^ ^]^ Theoretical calculations suggest that interfacing BSO with almost lattice‐matched LaInO_3_ (LIO) ($\delta =\frac{a_{\text{BSO}}-a_{\text{LIO}}}{a_{\text{BSO}}}\approx$ 0.24% lattice mismatch,^[^ ^22^, ^23^ ^]^) leads to the formation of a 2DEG inside the BSO for an SnO_2_/LaO terminated interface layer due to polar‐discontinuity doping,^[^ ^24^ ^]^ while for the BaO/InO_2_ interface termination a 2D hole gas inside the LIO is formed.^[^ ^25^ ^]^ Electrical transport measurements on these and other systems using BSO as a channel material validate the presence of a 2DEG, however, they suffer from freeze‐out of the 2DEG^[^ ^26^ ^]^ or need additional La doping of the BSO channel.^[^ ^27^, ^28^, ^29^ ^]^ These studies result in RT mobility values of 20–60 cm^2^ Vs^−1^ and leave a key subject of interest untouched, i.e., interface termination. A recent combined experimental and theoretical study indicates a preference for SnO_2_/LaO terminated interface formation in pulsed laser deposition (PLD) and MBE grown BSO/LIO heterostructures electrical properties of PLD grown samples are shown in ref. [29] and are depicted in Figure 3d–f and 4 indicated as [Pfü]. MBE grown samples of that study are shown in Figure 3a–d indicated as “mixed termination” with a LIO layer thickness of 3 nm.^[^ ^30^ ^]^ In comparison, refs. [31, 32] show that the intentional deposition of an SnO_2_ layer at the BSO/LIO interface results in a decrease of sheet resistance. However, charge carrier concentration and mobility remain behind the values of the abovementioned systems by one order of magnitude.

STO‐based 2DEGs^[^ ^9^ ^]^ have been demonstrated on STO substrates whose surface termination has been accurately controlled by chemical preparation.^[^ ^34^, ^35^ ^]^ Yet, the issue of controlled interface termination away from the substrate remains an outstanding challenge for 2DEGs formed at oxide interfaces.^[^ ^36^, ^37^ ^]^ The realization of a reproducible growth protocol for perovskite oxides, ensuring both the highest crystalline quality (maximizing electron mobility) and the most chemically sharp interfaces (maximizing charge transfer, hence carrier concentration), would open up for the insertion of 2DEG interfaces into intended positions in the device environment, since the substrate interface no longer participates.

While adsorption‐controlled MBE, by co‐deposition of cations A and B and self‐regulated stoichiometry due to desorption of the excess cation, has demonstrated the highest material quality of ABO_3_ perovskite thin films,^[^ ^19^, ^20^, ^38^, ^39^ ^]^ it lacks a good definition of the surface termination (AO or BO_2_) required for the heterostructure. In turn, the termination is best defined in a shutter‐controlled layer‐by‐layer growth,^[^ ^40^, ^41^, ^42^ ^]^ in which an AO and a BO_2_ monolayer (ML) are alternatingly deposited, yet coming at the expense of inevitably accumulating a net non‐stoichiometry due to the limited control on the provided A and B fluxes.

In the following, we identify control of the interface termination as a key ingredient for high‐mobility 2DEGs in BSO/LIO heterostructures. For this purpose, we demonstrate a growth protocol of the interface termination by sequentially changing from adsorption‐controlled MBE of BSO and LIO layers to layer‐by‐layer growth of their interfacial monolayers. A full set of electrical transport data verifies that our approach of interface termination results in BSO‐based 2DEGs with record RT mobility values up to 119 cm^2^ Vs^−1^—all achieved despite the lack of perfect lattice matching to the DyScO_3_ (DSO) substrate (δ ≈4.2% lattice mismatch), indicating the potential for even further improvement of this value. The employed approach, which can be generalized to almost all perovskite oxide interfaces, has the potential to empower the position of BSO/LIO and similar perovskite heterostructure systems within the field of 2DEG‐based high electron mobility transistors (HEMTs) and related devices.^[^ ^43^, ^44^ ^]^

## 2 Results and Discussion

### 2.1 Designing the Interface Termination

**Figure 1:**
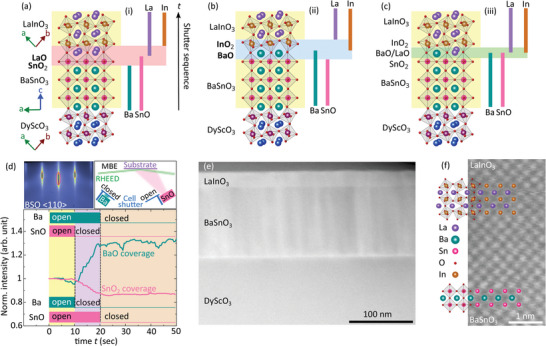
a–c) Sketch of the three different growth approaches to define the interface termination of a BSO/LIO heterostructure. The yellow shaded areas indicate co‐deposition growth in an adsorption‐controlled growth regime while the red (blue) shaded areas highlight interface termination using layer‐by‐layer growth. The vertical bars indicate the related shutter‐controlled supply of particle flux from the corresponding effusion cell. d) Normalized RHEED intensity of the specular spot (red tracer circle shown in the upper left corner) during BSO growth as a function of time followed by shutter‐controlled deposition (see sketch in the upper right corner) for the realization of SnO_2_ (magenta curve) versus BaO (blue curve) termination. e) Cross‐sectional scanning transmission electron microscopy (STEM) bright field image of the LIO/BSO/DSO heterostructure. f) Enlarged area of the BSO/LIO interface revealing coherent growth of the heterostructure. Atomistic models of BSO and LIO were superimposed to the STEM image using VESTA.^[^ ^33^ ^]^

To demonstrate the intentional control of the 2DEG formation by controlling the interface termination in BSO/LIO heterostructures, three distinctly different approaches to interface formation are compared during the transition from the growth of the BSO layer to the LIO layer on top. As schematically shown in **Figure** **1**a–c, these approaches are (i) the growth of a shutter‐controlled SnO_2_ monolayer (ML) followed by one ML of LaO, (ii) the growth of a shutter‐controlled BaO ML followed by one ML of InO_2_, or (iii) the absence of shutter‐controlled ML deposition. In all three approaches, the bulk of the BSO and LIO layers are individually grown by adsorption‐controlled co‐deposition to benefit from the associated low rate of point defect formation in this growth mode.^[^ ^19^, ^20^, ^39^, ^45^ ^]^

First, we demonstrate that single‐layer (AO or BO_2_) deposition following co‐deposition is possible. Thus, we assume interface termination can be realized. In Figure 1d, the BSO‐related normalized reflection high‐energy electron diffraction (RHEED) intensity of the specular spot during approaches (i) versus (ii) depicted in Figure 1a,b, respectively, is monitored as a function of BSO growth time. The yellow shaded area marks the final seconds of the BSO growth of a ≈100 nm thick BSO film on a DSO substrate by adsorption‐controlled co‐deposition (Ba and SnO shutters both open). The constant RHEED intensity likely indicates a stable, mixed BaO and SnO_2_ termination averaged over the macroscopic footprint of the RHEED beam on the growth front. Starting at *t* = 10 s (purple shaded area), either the Ba shutter was closed (whereas the SnO shutter remained open) for SnO_2_ termination [magenta curve, approach (i)], or the SnO shutter was closed (Ba shutter remained open) for BaO termination [blue curve, approach (ii)]. Growth of SnO_2_ on top (only SnO shutter open, red curve) leads to a decreasing RHEED intensity. Growth of BaO on top (only Ba shutter open, cyan curve), in contrast, leads to an increasing RHEED intensity in accordance with RHEED oscillations of this system.^[^ ^46^ ^]^ The brown shaded area marks the region where both cell shutters were closed to stabilize the surface before continuing with LIO deposition that starts either with one monolayer LaO [approach (i)] or InO_2_ [approach (ii)]. The continuously constant RHEED intensity after the intended species deposition suggests that these terminations of SnO_2_ and BaO are stable and remain on the surface. Growth details and discussion on deposition times can be found in the Experimental Section.

### 2.2 Structural Integrity

Scanning transmission electron microscopy (STEM) images verify the quality of the BSO/LIO heterostructure. On a larger length scale (Figure 1e), the DSO substrate, BSO layer, and LIO layers can be clearly distinguished. In addition, misfit dislocations at the DSO/BSO interface are revealed by their strain field. They arise from the significant lattice mismatch between DSO and BSO of ≈4.5% and result in threading dislocations within the BSO film that penetrate the LIO and likely limit the achievable electron mobility in the heterostructure. A coherent BSO/LIO interface region is shown in Figure 1f. The coherent growth of BSO/LIO heterostructure using approach (i) was also monitored by in situ RHEED (see Figure S1, Supporting Information).

Figure S3c,d (Supporting Information) shows the surface morphology measured by atomic force microscopy of the corresponding LIO/BSO heterostructure with nominally SnO_2_/LaO interface [approach (i)], and confirm single crystalline growth of individual BSO as well as coherently grown LIO layers by x‐ray diffraction scans, respectively. The surface exhibits information of substrate terrace formation (Figure S3a,b, Supporting Information), despite a total layer thickness of 130 nm. The heterostructures grown with the BaO/InO_2_ termination by approach (ii) and without intentional interface termination by approach (iii) (Figures S4 and S5, Supporting Information) show similar structural properties.

### 2.3 Interface‐Termination Design Determines Presence of 2DEG

Next, we show that the engineered interface terminations discussed above each result in drastically different electrical behaviour of the BSO/LIO samples. Capacitance–voltage (C–V) measurements, as described in ref. [29] and schematically illustrated in Figure S7a,b (Supporting Information), were performed on BSO/LIO heterostructures grown by all approaches (i), (ii), and (iii).

**Figure 2:**
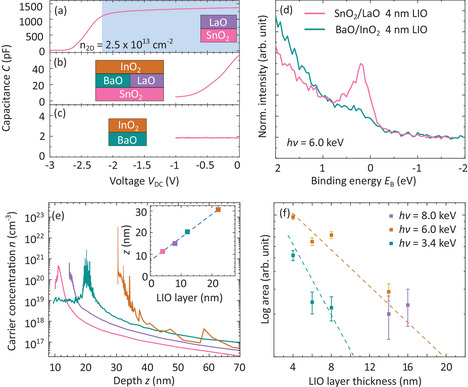
C–V measurement of the samples with a) SnO_2_/LaO interface termination, b) mixed interface termination, and c) BaO/InO_2_ interface termination as depicted in the sketch. From the blue shaded area in (a), the charge carrier accumulation at the interface was derived. d) Hard x‐ray photoelectron spectroscopy (HAXPES) valence band spectra, with the spectra magnified on the Fermi edge to elucidate the charge carriers at the interface for SnO_2_/LaO (red) and BaO/InO_2_ (cyan) interface termination for the samples with a 4 nm thick LIO layer. e) Charge carrier concentration profiles across the LIO/BSO interface derived from C–V measurements as shown in (a) using Equation (6) for different LIO layer thicknesses. Inset: position of the charge accumulation layer as a function of nominally deposited LIO layer thickness. f) Area dependence of HAXPES charge carrier signal at the Fermi edge shown in (d) as a function of LIO layer thickness for different photon energies *h*ν ‐ all samples contain the SnO_2_/LaO interface termination. The dashed cyan and brown lines depict the expected signal reduction due to LIO layer thickness and were derived from Sn 3d_5/2_ core levels collected at 3.4 keV and 6.0 keV, respectively.

While for the intended SnO_2_/LaO termination [(approach (i)] the measurements, shown in **Figure** **2**a, indicate charge carrier accumulation in the range of low 10^13^ cm^−2^ (blue shaded area), any mobile charge carriers are completely absent for the intended BaO/InO_2_ interface termination realized by approach (ii), as shown in Figure 2c. For BSO/LIO heterostructures without intentionally defined (“mixed”) interface termination [(approach (iii)], the C–V measurements indicate sample‐to‐sample variation fitting between the two above‐discussed scenarios (Figure 2b; Figure S7c, Supporting Information).

These results are independently corroborated by hard x‐ray photoelectron spectroscopy (HAXPES) measurements. The signal at the Fermi energy, i.e., at binding energy *E*_*B*_ = 0, in Figure 2d indicates free charge carriers in the BSO/LIO system. The recorded free carrier signal of a sample grown with shutter‐controlled SnO_2_/LaO interface termination [magenta solid line, approach (i)] is always larger compared to the signal from BaO/InO_2_ interface termination (cyan solid line, approach ii). Details on HAXPES measurements can be found in the experimental section as well as in the Supporting Information and related Figures S7d and S8 (Supporting Information).

C–V and HAXPES experiments of SnO_2_/LaO‐terminated BSO/LIO heterostructures grown by approach (i) with varying LIO layer thickness further confirm that the charge carrier accumulation is located at the interface. The peak position of the volume carrier concentration as a function of probing depth, shown in Figure 2e and obtained from the C–V measurements using Equation (6), reflects the distance of the charge carrier accumulation to the LIO surface. As shown in the inset of Figure 2e, it follows the nominal LIO layer thickness, suggesting accumulation at the LIO/BSO interface. A δ_off_ = 7 nm offset was applied to the data, which we attribute to a dielectric contamination layer accumulated at the Hg surface. In HAXPES measurements, the same results were achieved using different x‐ray energies in combination with different LIO layer thicknesses from 4 nm to 20 nm, shown in Figure 2f. The HAXPES spectra from which the area values at E_*f*_ were extracted are shown in Figure S2 (Supporting Information). As the photon energy is increased from 3.4 to 8.0 keV, the maximum inelastic mean free path (IMFP) of the photoelectrons increases from 4.95 to 10.1 nm.^[^ ^47^ ^]^ The probing depth is generally considered to be three times the IMFP (equivalent to detecting 95% of the total photoelectron signal), allowing for the detection of charge carrier accumulation at the BSO/LIO interface through thicker LIO layers.

Only when the photon energy is high enough to excite photoelectrons from the buried interface, it is possible to detect the charge accumulation feature in the HAXPES data.

Since the signal of charge carriers at the *E*_*F*_ decreases with increasing LIO layer thickness in the same manner as the signal of Sn 3d core‐level (cyan and brown dashed lines at 3.4 and 6.0 keV, respectively), we attribute the presence of the charge carriers to the interface of the BSO/LIO heterostructure. We rule out the presence of a broader doped region inside the BSO layer by comparison to Figure 2d, which compares two identical heterostructures which only differ in their interface termination.

Interestingly, the C–V measurements also demonstrate that full depletion of the charge accumulation is possible—a mandatory ingredient for further processing toward transistor applications. Moreover, applied voltages of 3–10 V for LIO layer thicknesses in the range of 4–24 nm correspond to high breakdown field strengths of the LIO layers in the range of 2.7–3.2 MV cm^−1^ (considering the δ_off_ = 7 nm offset).

### 2.4 2DEG Transport Properties

**Figure 3:**
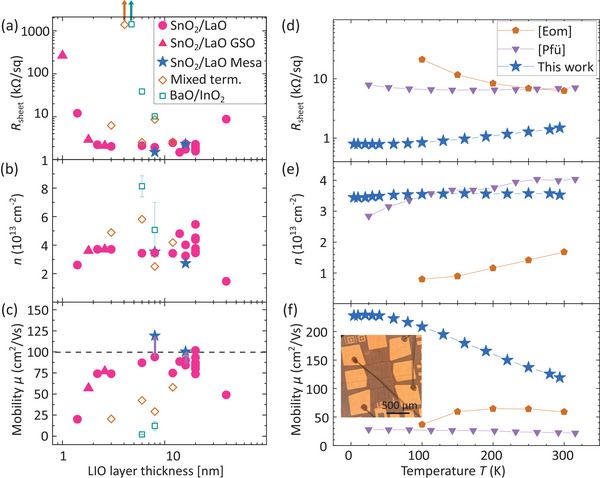
Electrical transport properties: a) sheet resistance, b) charge carrier concentration, and c) mobility as a function of LIO layer thickness and interface termination [approach (i), (ii), and (iii)] at RT. All data points correspond to heterostructures grown on DSO substrates except for the magenta triangles which correspond to heterostructures grown on GdScO_3_ (GSO) substrates. d–f) Temperature‐dependent measurement and comparison to other BSO based 2DEGs labeled as follows: [Eom],^[^ ^26^ ^]^ [Pfü].^[^ ^29^ ^]^

The electron transport properties in the as‐grown heterostructures with different LIO layer thicknesses and designed interface termination were determined by van–der–Pauw–Hall (vdP) measurements. Details on contact deposition and lithographically defined structures (**Figure** **3**f) can be found in the experimental section. In Figure 3a–c, the RT sheet resistance, charge carrier concentration, and mobility, respectively, are shown for different LIO layer thicknesses, different substrates, and differently designed interface terminations.

We find that the LIO/BSO/DSO (read top to bottom) heterostructures with designed SnO_2_/LaO interface (magenta circles) generally exhibit the lowest sheet resistance values (≈2 kΩ/sq) and charge carrier densities of ≈3.5 − 5 × 10^13^ cm^−2^, resulting in mobility values in the range of 75–100 cm^2^ Vs^−1^ for LIO thicknesses from 2 to 20 nm. In addition, similar values were achieved using GdScO_3_ (GSO) substrates (magenta triangles), demonstrating that our approach is not limited to the choice of substrate. The increase of sheet resistance and drop of charge carrier concentration and mobility for heterostructures with decreasing LIO layer thickness below 2 nm is in accordance with theoretical predictions^[^ ^25^ ^]^ corresponding to ionic displacement and octahedral tilt in both materials that screen the electric field induced by the polar discontinuity. Such critical thickness for 2DEG formation has also been observed in PLD‐grown samples.^[^ ^48^ ^]^ Using a lithographically‐defined, mesa‐isolated vdP structure, record mobilities in perovskite oxide 2DEGs up to 119 cm^2^ Vs^−1^ at RT were measured (blue asterisks in Figure 3a–c), which is by far the highest value for as‐grown samples reported in the literature to the best of our knowledge.

In contrast, significantly higher sheet resistances were measured for samples grown with the designed BaO/InO_2_ interface (empty cyan squares). Hall measurements indicate an increased charge carrier concentration and, thus, low mobilities. The high apparent Hall charge carrier concentration contradicts the results of C–V measurements. It is considered to be an artefact that results from non‐ideal transport along percolative paths causing a drop in measured Hall‐voltage. This is further elaborated in the Supporting Information and related Figure S6 (Supporting Information).

Electron transport properties of BSO/LIO heterostructures without designed interface termination (empty brown diamonds) fluctuate between those of heterostructures with designed SnO_2_/LaO interface termination and BaO/InO_2_ interface termination in line with the C–V measurements shown in Figure S7c (see Supporting Information), and are thus consistent with an uncontrolled, fluctuating, possibly laterally inhomogeneous/mixed interface termination for these samples.

The temperature dependence of transport properties of a SnO_2_/LaO interface terminated 120 nm/20 nm BSO/LIO sample shown in Figure 3d–f qualitatively agrees with the behavior of other known 2DEGs: A decreasing sheet resistance with decreasing temperature from 300 to 4 K can be observed in Figure 3d. The comparably constant electron concentration in this temperature range, visible in Figure 3e, is a further indication of charge accumulation (a degenerate system that cannot freeze out). The related electron mobility in Figure 3f increases with decreasing temperature, in agreement with the reduction of phonon scattering, and saturates at 228 cm^2^ Vs^−1^ below 50 K—likely due to the presence of threading dislocations originating from the DSO/BSO interface. The temperature‐dependent transport properties (cf. Figure 3d–f) of previously published work show significantly lower electron mobility values (ref. [29]) or indication of a freeze‐out of the carrier in ref. [26], which is atypical for a real 2DEG.

**Figure 4:**
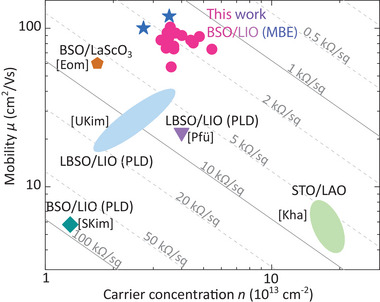
Comparison of RT 2DEG sheet resistance, charge carrier concentration, and mobility to other oxide systems. The magenta circles and blue asterisks indicate 2DEG mobility values and carrier concentration of our MBE grown samples. Mobility values and carrier concentration of 2DEG systems of other groups are shown as colored icons or ellipses (in case of multiple values). The two layers of the 2DEG forming heterostructure are written next to the icons. The reference data is indicated by square brackets with the following relation: [Kal],^[^ ^49^ ^]^ [Eom],^[^ ^26^ ^]^ [UKim],^[^ ^50^ ^]^ [Pfü],^[^ ^29^ ^]^ [SKim],^[^ ^32^ ^]^ [Kha].^[^ ^14^ ^]^ The grey lines (solid and dashed) indicate the values of the sheet resistance.

Hence, the MBE‐grown LIO/BSO samples of the present work with a shutter‐controlled SnO_2_/LaO interface termination stand out by a factor of two or more compared to published RT transport properties of other BSO‐related 2DEGs (**Figure** **4**) in terms of high charge carrier concentration, high mobility and low sheet resistance (both crucial for HEMT applications). STO‐based systems, that can have higher charge carrier concentration are clearly outperformed in terms of application‐relevant RT electron mobility. We note that while our BSO/LIO‐based 2DEGs achieve the highest RT electron mobility values to date as well as comparable interfacial carrier concentration to other BSO‐based systems, our measured carrier densities do not reach the carrier concentration limit of approximately 3 × 10^14^ cm^−2^ as predicted by the polar catastrophe model.^[^ ^25^ ^]^ Although we expect that further growth optimization such as better lattice matching to the underlying substrate could yield even higher values, previous work on similar systems suggest that additional mechanisms lowering the achievable carrier concentration, such as polarization effects in the heterostructure,^[^ ^25^, ^28^, ^51^ ^]^ also could be at play.

## 3 Summary and Conclusion

In summary, we have experimentally identified the interface engineering as a key ingredient for high‐mobility 2DEGs in BSO/LIO heterostructures. We have furthermore established a path to realizing a specific interface termination while ensuring high layer quality for MBE‐grown BSO/LIO samples by combining an adsorption‐controlled co‐deposition with a layer‐by‐layer growth of the interfacial monolayers. Both C‐V and HAXPES measurements show a drastically enhanced charge accumulation at the BSO/LIO interface with engineered SnO_2_/LaO interface termination compared to BaO/InO_2_ and mixed interface terminations, in agreement with theoretical predictions.^[^ ^25^ ^]^ In such samples, electrical transport measurements revealed RT mobility values up to 119 cm^2^ Vs^−1^, sheet resistance values down to 1.8 kΩ sq^−1^ and a carrier concentration of *n* ≈3.6 × 10^13^ cm^−2^.

Further mobility improvement can be expected for samples with reduced dislocation density and improved interface roughness. Notwithstanding, our results already surpass existing BSO‐based 2DEGs by a factor of at least two in terms of increased mobility and decreased sheet resistance. We further note that the high breakdown field strengths in the range of 2.7–3.4 MV cm^−1^ in the LIO layer make it a suitable dielectric that allows full depletion of the 2DEG. All this, in combination with high charge carrier densities, establishes BSO/LIO 2DEGs as promising building blocks for perovskite‐based HEMTs with unprecedented performance. Additionally, their potential for monolithic integration with further perovskite‐based functional layers opens up for realization of novel oxide electronic devices such as high‐electron mobility ferroelectric FETs.^[^ ^44^, ^52^, ^53^ ^]^ Lastly, this approach of combining co‐deposition with layer‐by‐layer growth to realize a specific interface termination is not a unique feature of the BSO/LIO heterostructure but can be applied to any other perovskite system.

## 4 Experimental Section

### MBE Growth

For the growth of the BSO/LIO heterostructure, DSO substrates were used. Details on substrate preparation are described in ref. [45]. The BSO was grown using a mixture of SnO_2_ and Sn as source material resulting in a SnO suboxide flux at comparably low cell temperatures in the range of 740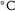 – 850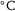. Details on the suboxide sources can be found in ref. [54]. Stoichiometric LIO layers were grown according to the growth conditions evaluated in ref. [45]. For the present study, Ba, SnO, In, and La cell fluxes were chosen in such a way, that substrate temperature, oxygen flux and plasma power were kept constant at 835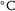 (measured by a pyrometer), 0.065 sccm, and 200 W, respectively. To keep the plasma source running, 0.2 sccm Ar supporting gas was added to the gas flow. More details can be found in the Supporting Information. As a consequence of the adjustment of the B‐cation fluxes and growth parameters, the growth of the BSO/LIO heterostructure and interface termination reduces to a simple cell shutter sequence.

### Interface Termination

Assuming that the BSO growth front during Co‐deposition exhibits a SnO_2_/BaO termination ratio in the range of 1:2 ‐ 2:1. To achieve full coverage with the same termination up to 0.67 ML of the intended species needs to be supplied. For a BSO growth rate of 1.8 nm min^−1^, the time for a BaO or SnO_2_ layer to form would be ≈14 s. Consequently, shutter times in the range of 9–10 s seem to be suitable for the realization of majority terminations. For The LIO growth, LaO or InO_2_ shutter was opened for ≈15 s (a growth rate of 1.2 nm min^−1^ corresponds to LaO or InO_2_ monolayer deposition within 20 s. Thus, 3/4 of the interface termination is defined before starting co‐deposition growth that completes interface termination and realizes LIO growth.

### TEM

For TEM analysis of the samples, an aberration‐corrected FEI Titan 80–300 operating at 300 kV was used. For recording scanning TEM (STEM) images, a high‐angle annular dark‐field (HAADF) detector and a camera length of 195 mm was used. TEM cross‐sectional samples were prepared and analyzed along the [110] projection of the DSO substrate. Analyzed heterostructures are projected along the [110] direction of DSO and [100] direction of BSO.

### Processing Transport Structures

For electrical characterization, Ti/Au contacts with a thickness of 20/100 nm were deposited at the corners (as well as at the edges) of the 5×5 mm^2^ samples by sputtering using a shadow mask. The diameter of the contacts was 0.5 mm at the edges and 1 mm at the corners. Further, structures with a more precise geometry were realized by photolithography using a shadow mask. For mesa definition, wet chemical etching was done (2 min sample etching in HF:HNO_3_:H_2_O: 2/10:4:100 mL at an etching rate of 70 nm min^−1^). For sample contacts, Ti/Au 10/90 nm were deposited using an electron beam evaporator. For the lift‐off of metal on resist acetone was used.

### Electrical Transport

The samples were electrically investigated using the vdP method.^[^ ^55^, ^56^ ^]^ The following formulas were used to extract sheet resistance *R*_sheet_ and charge carrier concentration $n=\frac{1}{eR_{H}}$, from which mobility $\mu =\frac{R_{H}}{\rho }$ was derived:

(1) $$\begin{array}{c} R_{\text{sheet}}=\frac{\pi }{\ln2}\frac{R_{\text{(AB,DC)}}+R_{\text{(BC,AD)}}}{2}\cdot f_{\text{vdP}} \end{array}$$

The 4‐terminal resistance is defined by *R*_(AB, DC)_ = *I*_AB_/*U*_DC_ (Figure 3a), and *f*_vdp_ is a form factor that takes the sample geometry into account, and that can be derived from the expression:^[^ ^55^, ^56^ ^]^

(2) $$\begin{array}{c} \cosh\left\{\frac{R_{\text{(AB,DC)}}/R_{\text{(BC,AD)}}-1}{R_{\text{(AB,DC)}}/R_{\text{(BC,AD)}}+1}\cdot \frac{\ln2}{f_{\text{vdP}}}\right\}=\frac{1}{2}\cdot \exp\frac{\ln2}{f_{\text{vdP}}} \end{array}$$

Note that Equation (2) can only be solved implicitly.

Further, the Hall resistance in vdP configuration is given by:^[^ ^55^, ^56^ ^]^

(3) $$\begin{array}{c} R_{H}=\frac{1}{B_{z}}\Delta R_{\text{(BD,AC)}} \end{array}$$

where Δ*R*_(BD, AC)_ is the resistance change of *R*_(BD, AC)_ due to the magnetic field *B*_*z*_ as shown in Figure 4b.

### Capacitance–Voltage Measurements

C–V measurements were performed using a Keithley 4200 measurement device connected to an Hg probe system with a 0.3 mm diameter disc‐shaped gate contact. A 10 kHz, 50 mV rms *ac* voltage was used and the capacitance was extracted using the series circuit model. Figure S7a,b (Supporting Information) show a top and side view of a LIO/BSO heterostructure with two mercury probes being in contact with the sample surface, respectively. In Figure S7b (Supporting Information) the equivalent circuit model is drawn into the depicted LIO/BSO heterostructure. Since the capacitance *C*_2_ ≫ *C*_1_ due to the different area size (the area of *C*_1_ has a size of *A*_1_ = 7.3 × 10^−2^ mm^2^, the area of *C*_2_ has a size of *A*_2_ ≈19 × *A*_1_), the total capacitance is given by:

(4) $$\begin{array}{c} \frac{1}{C}=\frac{1}{C_{1}}+\frac{1}{C_{2}}\approx \frac{1}{C_{1}} \end{array}$$

The samples were analyzed using a serial model at an *ac* frequency in the range of 10–50 kHz and using ϵ_*r*_ of 21 for the calculation of the capacitance.

In addition, the 2D‐charge carrier concentration can be calculated according to:

(5) $$\begin{array}{c} n_{\text{2D}}=\frac{C\Delta V_{\text{dc}}}{A_{1}e} \end{array}$$

where Δ*V*_dc_ is the *dc* voltage difference for which *C* is constant (blue shaded area in Figure 3c), and *A*_1_ is the size of the small Hg contact.

The charge carrier concentration as a function of the depth can be derived from the slope of 1/*C* ^2^ as a function of *V*_dc_:

(6) $$\begin{array}{c} n\left(z\right){|}_{z=W}=-\frac{2}{\varepsilon_{r}\varepsilon_{0}}{\left[\frac{d\left(\frac{1}{C^{2}}\right)}{dV_{\text{dc}}}\right]}^{-1} \end{array}$$

Note that the relation between *C* and *W* is given by:

(7) $$\begin{array}{c} C=\frac{\varepsilon_{r}\varepsilon_{0}A}{W} \end{array}$$

### Hard X‐Ray Photoelectron Spectroscopy (HAXPES)

HAXPES data were collected at beamline P22 at PETRAIII, German Electron Synchrotron DESY in Hamburg, Germany.^[^ ^57^ ^]^ Three photon energies were used, including 3.4, 6.0, and 8.0 keV. A Si (311) double‐crystal monochromator was used to achieve the two higher photon energies. A double channel‐cut post‐monochromator employing a Si(111) and a Si(220) channel‐cut crystal pair was used to select 3.4 keV. The beamline employs a Phoibos 225HV analyzer (SPECS, Berlin, Germany), which was set up in the small area lens mode with a slit size of 3 mm for the collection of spectra. Spectra were collected using a pass energy of 30 eV. The total energy resolution for the three photon energies in this setup was determined based on the 16/84% Fermi edge (*E*_*F*_) width of a polycrystalline gold foil.^[^ ^58^ ^]^ The resulting total resolutions were 215, 249, and 260 meV for 3.4, 6.0, and 8.0 keV, respectively. All experiments were conducted in grazing incidence geometry (⩽5°). Survey scans and core‐level (CL) spectra are shown in Figure S8 (Supporting Information). From all recorded CL spectra, a Shirley background was subtracted. The spectra were normalized to the sum of the Sn 3d_5/2_ and In 3d_5/2_ core‐level area and either aligned to the internal 50% value at the Fermi edge or to *E*_*F*_ (Au) in case of absence of free carriers at *E*_*F*_. For the investigation of the 2DEG area, an additional linear background was subtracted.

## Conflict of Interest

The authors declare no conflict of interest.

## Supporting information

Supporting Information

## Data Availability

The data that support the findings of this study are available from the corresponding author upon reasonable request.
