# Supplementary material for: Enabling 2D Electron Gas with High Room‐Temperature Electron Mobility Exceeding 100 cm2 Vs−1 at a Perovskite Oxide Interface
Source: Adv Mater. 2024 Oct 22;36(50):2409076. doi: 10.1002/adma.202409076 (PMC11636175; doi:10.1002/adma.202409076)
Supplement: Supplementary file 1 — Supporting Information [file ADMA-36-2409076-s001.pdf]

ADVANCED MATERIALS

Supporting Information

for *Adv. Mater.*, DOI 10.1002/adma.202409076

Enabling 2D Electron Gas with High Room-Temperature Electron Mobility Exceeding 100 cm^2 Vs^{-1} at a Perovskite Oxide Interface

Georg Hoffmann, Martina Zupancic, Aysha A. Riaz, Curran Kalha, Christoph Schlueter, Andrei Gloskovskii, Anna Regoutz, Martin Albrecht, Johanna Nordlander and Oliver Bierwagen*

1. SUPPLEMENTARY INFORMATION

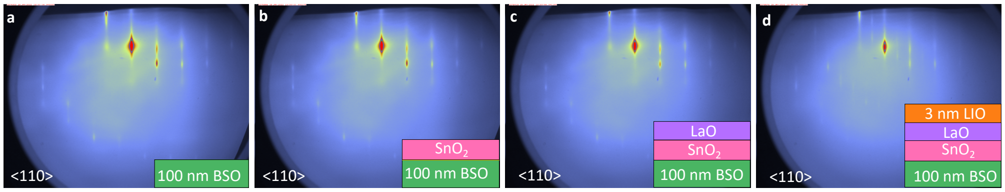

Fig. S1. **a–d** RHEED images indicating continuity of the crystal structure during shutter-controlled interface formation: **a** RHEED image along the BSO <011> azimuth of a 100 nm thick BSO film grown on a $DyScO_3$ substrate using Ba and SnO co-deposition. **b** RHEED image of nominally SnO_2 terminated BSO surface by closing the Ba shutter 10 s before the SnO shutter. **c** Realizing of SnO_2/LaO interface termination by opening only the La shutter 15 s before the In shutter. **d** LIO growth using co-deposition of La and In in an adsorption-controlled growth regime.

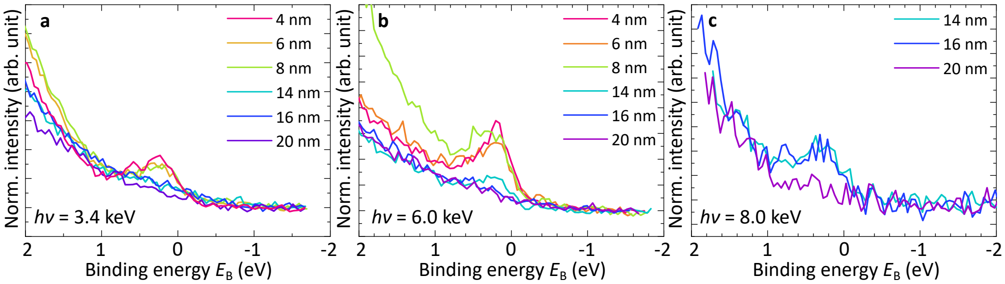

Fig. S2. HAXPES measurements of charge carriers at the Fermi edge for different photon energies $h\nu$ as well as LIO layer thicknesses - all samples contain the SnO_2/LaO interface termination. HAXPES scans around the Fermi edge of BSO/LIO heterostructures at photon energies of **a** 3.4 keV, **b** 6.0 keV and **c** 8.0 keV. The legend depicts the LIO layer thickness of the samples. The signal of free charge carriers is measured as an increase in the intensity of the states below the Fermi edge. With an increase in the photon energy, the LIO layer thickness through which the charge carriers can be detected increases from 8 nm at 3.4 keV to 16 nm at 8.0 keV, thus demonstrating that the charge carriers are not a surface effect but located at the BSO/LIO interface.

A. Growth of $BaSnO_3$ and $LaInO_3$

BSO and LIO are grown in an adsorption-controlled growth,[1, 2] i.e. growth in excess of the more volatile B-cation. However, for both LIO and BSO, the growth window is rather narrow (± 2 °C in the cation fluxes). The Ba, La, and In cell temperatures were in the range of 580 °C - 630 °C, 1490 °C - 1550 °C, and 700 °C - 705 °C, respectively. The resulting growth rates of BSO and LIO were in the range of 1.8-2.1 nm/min, and 1.1-1.4 nm/min. Note that SnO and In were given in a slight excess (ratio of 1/1.5 - 1/3 to Ba and La, respectively) to ensure the adsorption-controlled growth. After growth, the oxygen supply was stopped when the substrate temperature was below 800 °C to avoid unintentional incorporation of Si into the films due to the quarz cavity used to generate the plasma.

B. Substrate preparation and sample analysis

The DSO samples were annealed in a tube furnace with a quartz tube at 1050 °C for 6 h. Additionally, an oxygen gas flow of ≈ 250 sccm was applied for the reconstruction of the DSO surface. The temperature ramp rates were set to 20 °C/min for heating up and cooling down. Figure S3**a** shows the DSO surfaces after the substrate preparation. It is assumed that the surfaces are singly terminated as indicated by the profiles of DSO shown in Figure S3**b**. According to the chosen annealing parameters for DSO, ScO_2 (B-site) termination is assumed.[3]

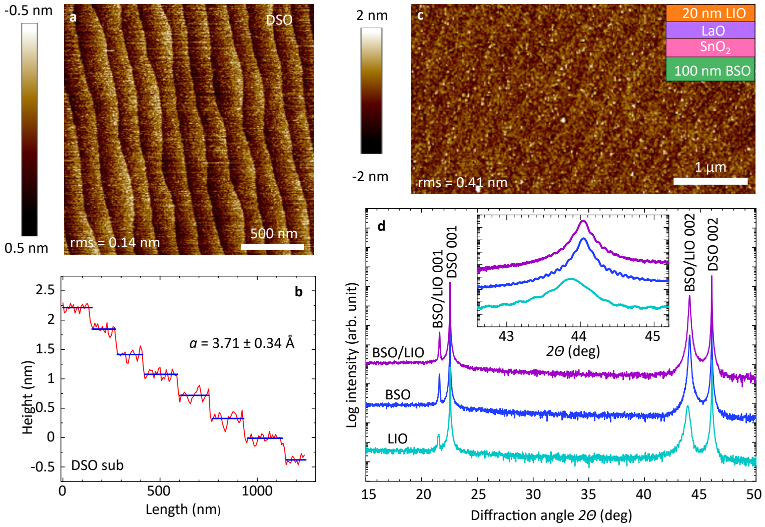

Fig. S3. **a** DSO substrate surface after preparation procedure. **b** shows the corresponding height profile derived from line scans. The average step edge height indicates single surface termination.**c** Atomic force microscopy image of the grown heterostructure and interface design as depicted in the sketch in the upper right corner. **d** X-ray diffraction symmetric $2\Theta - \omega$ scan for a grown LIO/BSO heterostructure (16/110 nm) with interface design as well as for single BSO (110 nm) and LIO (60 nm) films, all grown on DSO substrates. Thickness fringes of the 002 reflex shown in the inset reveal the presence of sharp interfaces.

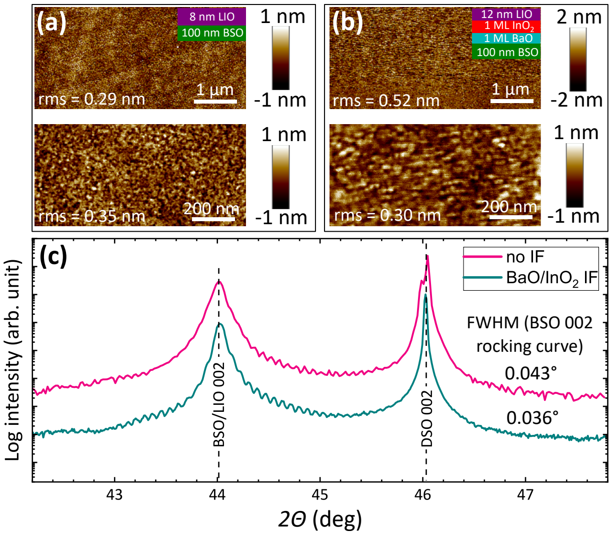

Fig. S4. AFM images of a sample without specific interface termination **a** and with BaO/InO_2 interface termination **b**. **c** XRD $2\Theta - \omega$ scan showing the BSO/LIO 002 reflection and the DSO substrate 002 reflection. Thickness fringes and FWHM values of the BSO 002 rocking curve indicate high crystalline quality.

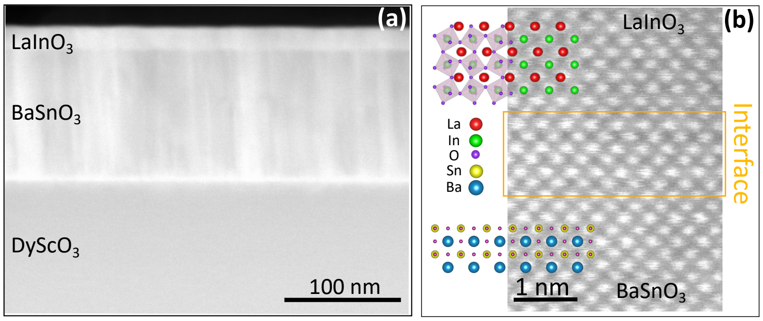

Fig. S5. STEM images of a LIO/BSO/DSO heterostructure **a** and coherent LIO/BSO interface **b** without any preferential interface termination. Atomistic models of LIO and BSO were superimposed on the image. The orange frame indicates the transition region from BSO to LIO.

Figure S4**a** and S4**b** show AFM images of BSO/LIO heterostructures without any preferential interface termination and BaO/InO_2 interface termination, respectively. The root mean square (rms) values are in the same range as the ones for SnO_2/LaO terminated heterostructures. Further, Figure S4**c** shows a symmetric XRD $2\Theta - \omega$ scan of the corresponding samples in Figure S4**a** and S4**b**. Also here, thickness fringes and FWHM values of BSO 002 rocking curves are comparable to the ones for SnO_2/LaO interface termination. In addition, Figure S5 shows a STEM image of a BSO/LIO heterostructure that has no specific interface termination. As for the STEM analysis of the sample with a SnO_2/LaO interface termination in the main paper, the crystallinity of the BSO and LIO layers (Figure S5**a**) as well as the coherent growth of LIO on BSO is confirmed (Figure S5**b**). Since the morphology of the samples grown according to approaches (i), (ii), and (iii) depicted in Figure 1**a** of the main manuscript is almost identical, we exclude the hypothesis, that variations of electrical transport properties are caused by morphological changes, but must be a direct consequence of the interface termination.

C. Discussion of Hall data

Due to finite interfacial roughness and growth conditions, the interface termination can vary between a total SnO_2/LaO termination and a BaO/InO_2 termination (Figure S6). While layer-by-layer interface growth tends to be close to single terminated interfaces, the termination contribution is completely different and unpredictable in the case of BSO and LIO co-deposition. Assuming still both interface terminations with a minority of the 2DEG hosting SnO_2/LaO interface termination, the prerequisites of vdP measurements are not given any more (i.e., homogeneous film without holes). Instead, electrons are now travelling only in the SnO_2/LaO interface areas along percolating (and by this longer) pathways to make it to the hall electrodes. Due to the longer pathway, charge carriers are scattered more often and no longer contribute to the Hall signal (a potential difference that only arises because of the presence of charge carrier accumulation at one of the electrodes due to the Lorentz force). While along the applied electric field, this leads to an increased sheet resistance in accordance with the observations, the Hall voltage is reduced resulting in a diminished Hall resistance and artificially increased CCD.

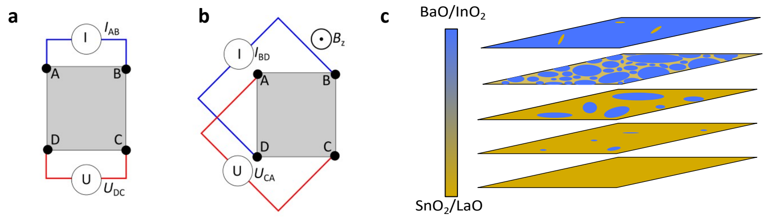

d

Sheet resistance (kΩ)	1 ML InO_2	0 ML LaO	1/2 ML LaO	1 ML LaO
1 ML BaO	-			
0 ML SnO_2		24		3.6
1/2 ML SnO_2			1.8	1.6
1 ML SnO_2		2.9		1.3 - 2.3

e

Mobility (cm^2/Vs)	1 ML InO_2	0 ML LaO	1/2 ML LaO	1 ML LaO
1 ML BaO	-			
0 ML SnO_2		2		26
1/2 ML SnO_2			81	96
1 ML SnO_2		67		92 - 116

f

CCD ($10^{13} cm^{-2}$)	1 ML InO_2	0 ML LaO	1/2 ML LaO	1 ML LaO
1 ML BaO	-			
0 ML SnO_2		16		6.6
1/2 ML SnO_2			4.3	4.0
1 ML SnO_2		3.2		2.8 - 4.5

Fig. S6. Illustration of transition from SnO_2/LaO to BaO/InO_2 interface termination. Since for areas with BaO/InO_2 termination, no electron transport is predicted, longer pathways and additional scattering are present in the case of mixed interface terminations.

D. Discussion of 2DEG identification

Figure S7**a** and S7**b** give insight into C-V measurement setup. Figure S7 shows an additional data set for a sample without preferred interface termination (third panel) as well as the Dissipation factor (black solid line) with respect to the right axis. Figure S7**d** illustrates the HAXPES measurement setup with three different photon energies. Figure S7**e** shows the 2DEG signal at the Fermi edge for samples with SnO_2/LaO and BaO/InO_2 interfaces for different LIO layer thicknesses written in the legend. Comparison of the data attributes an increased charge carrier accumulation for samples with SnO_2/LaO interface.

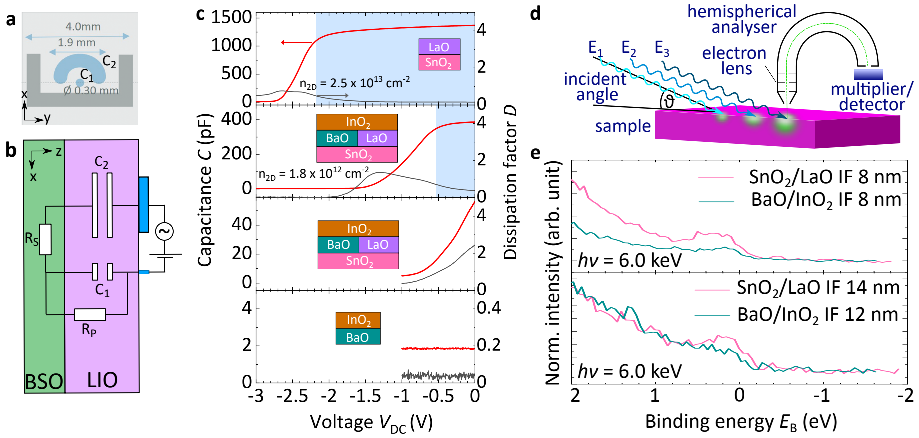

Fig. S7. **a** and **b** Schematic of contact geometry for C–V measurements from the top and the side, respectively, including equivalent current circuit. Blue marks the Hg contacts, light and dark grey mark the size of the samples and the vacuum pad, respectively. **c** C-V measurement (solid red line) with respect to the left axis of the samples with different or no specific interface design as depicted in the sketch. From the area within the blue-shaded region, the charge carrier accumulation at the interface can be derived. The dissipation factor of the measurements is shown as a solid, black line with respect to the right axis. **d** Illustration of X-ray photoelectron spectroscopy (PES) measurements with a variable photon energy source. **e** Hard X-ray photoelectron spectroscopy (HAXPES) measurements of charge carriers at the interface for SnO_2/LaO (red) and BaO/InO_2 (green) interface termination for samples with different LIO layer thicknesses [8 nm, and 12(14) nm].

E. Discussion of HAXPES survey scan and core-level data

Figure S8**a** shows representative survey scans of samples with SnO_2/LaO interface termination at 6.0 keV. The LIO layer thickness is written on top of the individual survey scan. All measured core levels can be attributed to BSO and LIO except a minor peak at 283 eV which corresponds to C 1*s* demonstrating the quality of the samples. The core-level spectra shown in Figure S8**b**–**f** reflect three aspects: (I) as LIO layer thickness increases La $3d_{3/2}$ and In 3*d* CL intensities increase, (II) with increasing LIO layer thickness, Ba $3d_{5/2}$ and Sn 3*d* signal decreases, and (III) O 1*s* signal remains approximately constant.

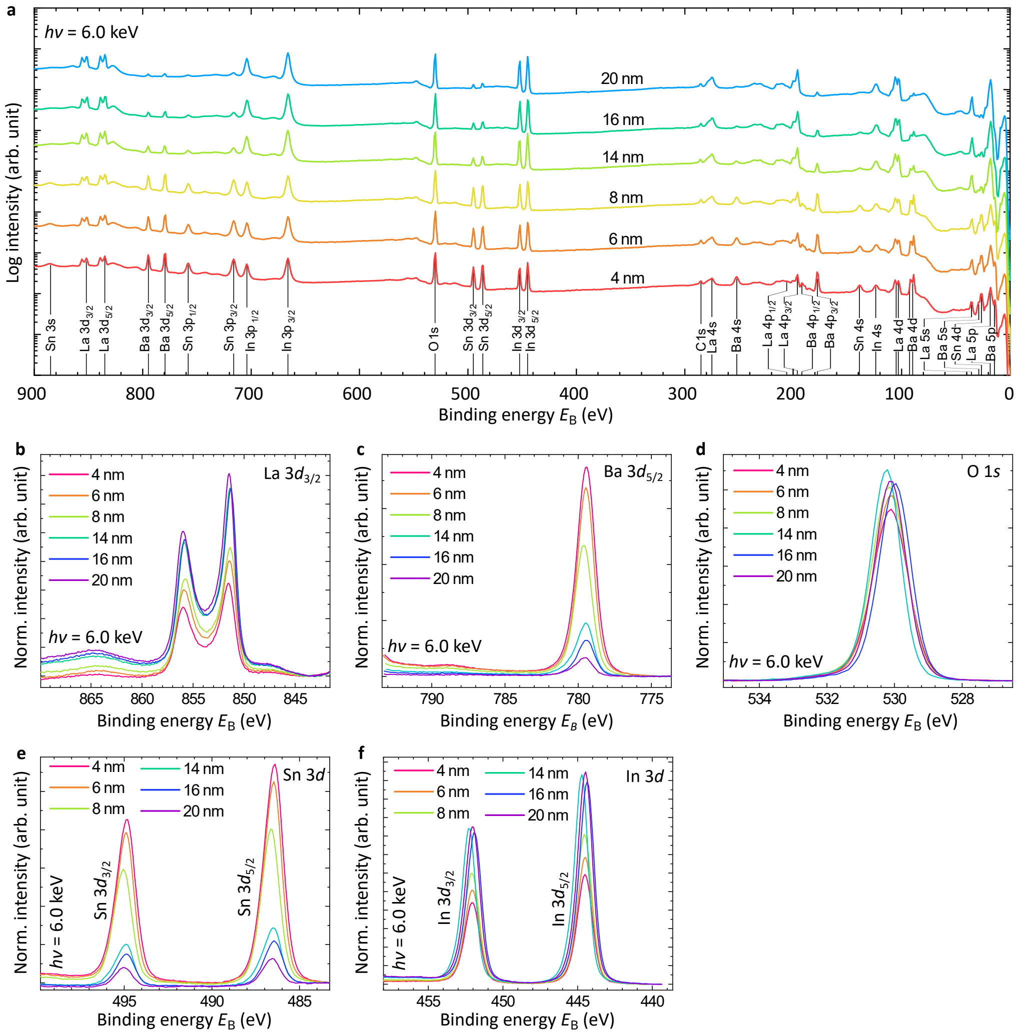

Fig. S8. **a** Representative HAXPES survey spectra of BSO/LIO heterostructures with varying LIO layer thickness from 4 nm to 20 nm written on top of the recorded spectra. The spectra were recorded at 6.0 keV and separated by an offset for better visualisation. Black vertical lines identify the core-level positions. **b**–**f** core-level spectra collected at 6.0 keV across the sample range. The spectra displayed are **b**: La $3d_{1/2}$, **c**: Ba $3d_{5/2}$, **d**: O $1s$, **e**: Sn $3d$, and **f**: In $3d$.

REFERENCES

1. H. Paik, Z. Chen, E. Lochocki, *et al.*, "Supplementary material: Adsorption-controlled growth of La-doped $BaSnO_3$ by molecular-beam epitaxy," APL Mater. **5**, 1–10 (2017).
2. G. Hoffmann, M. Zupancic, D. Klimm, *et al.*, "Adsorption-controlled plasma-assisted molecular beam epitaxy of LaInO 3 on DyScO 3 (110): Growth window, strain relaxation, and domain pattern," Phys. Rev. Mater. **7**, 084605 (2023).
3. R. Dirsyte, J. Schwarzkopf, G. Wagner, *et al.*, "Thermal-induced change in surface termination of $DyScO_3$(110)," Surf. Sci. **604**, L55–L58 (2010).
